# Oral microbe-host interactions: influence of β-glucans on gene expression of inflammatory cytokines and metabolome profile

**DOI:** 10.1186/s12866-017-0946-1

**Published:** 2017-03-07

**Authors:** Viviam de Oliveira Silva, Luciano José Pereira, Ramiro Mendonça Murata

**Affiliations:** 1 Herman Ostrow School of Dentistry, Division of Periodontology Diagnostic Sciences, Dental Hygiene & Biomedical Science, University of Southern California, Los Angeles, CA USA; 2 Department of Veterinary Medicine, Physiology and Pharmacology Area, Federal University of Lavras, Lavras, Minas Gerais Brazil; 3 Department of Health Sciences, Physiology Area, Federal University of Lavras,Lavras, Minas Gerais, Brazil; 4 School of Dental Medicine, Department Foundational Sciences, East Carolina University, 1851 MacGregor Downs Road, Greeville, NC 27834-4354 USA; 5 Brody School of Medicine, Department of Microbiology and Immunology, East Carolina University, Greenville, NC USA

**Keywords:** *Aggregatibacter actinomycetemcomitans*, Periodontal disease, Host response, Keratinocyte, Fibroblast, Immunomodulation

## Abstract

**Background:**

The aim of this study was to evaluate the effects of β-glucan on the expression of inflammatory mediators and metabolomic profile of oral cells [keratinocytes (OBA-9) and fibroblasts (HGF-1) in a dual-chamber model] infected by *Aggregatibacter actinomycetemcomitans*. The periodontopathogen was applied and allowed to cross the top layer of cells (OBA-9) to reach the bottom layer of cells (HGF-1) and induce the synthesis of immune factors and cytokines in the host cells. β-glucan (10 μg/mL or 20 μg/mL) were added, and the transcriptional factors and metabolites produced were quantified in the remaining cell layers and supernatant.

**Results:**

The relative expression of interleukin (IL)-1-α and IL-18 genes in HGF-1 decreased with 10 μg/mL or 20 μg/mL of β-glucan, where as the expression of PTGS-2 decreased only with 10 μg/mL. The expression of IL-1-α increased with 20 μg/mL and that of IL-18 increased with 10 μg/mL in OBA-9; the expression of BCL 2, EP 300, and PTGS-2 decreased with the higher dose of β-glucan. The production of the metabolite 4-aminobutyric acid presented lower concentrations under 20 μg/mL, whereas the concentrations of 2-deoxytetronic acid NIST and oxalic acid decreased at both concentrations used. Acetophenone, benzoic acid, and pinitol presented reduced concentrations only when treated with 10 μg/mL of β-glucan.

**Conclusions:**

Treatment with β-glucans positively modulated the immune response and production of metabolites.

## Background

β-glucans from yeast have been used extensively as protective substances against infections with potent effects on the innate and adaptive immune responses. β-glucans are non-starch polysaccharides that make up structural cells of plants and microorganisms [1]. The cell wall of *Saccharomyces cerevisiae* is an important source of β-glucans and these represents about 50–60% of yeast [2]. The protective effect of these compounds has been demonstrated in experimental infection [3]. Additionally, there are reports that these substances modulate allergy symptoms [4] and have anticancer properties [5, 6]. Many hypotheses have been put forward to explain the effects of β-glucans. Such compounds can act by inhibiting the adhesion of pathogens to epithelial tissues of the digestive tract by blocking carbohydrate-binding adhesins on bacteria; they stimulate the immunocompetent cells in Peyer’s patches and the consecutive activation of mechanisms of innate and adaptive immune defense; further, by adsorption of mycotoxins in food (when linked to the diet) β-glucans inhibit their toxic activity [2].

However, its effects on periodontal inflammation are still poorly studied. Periodontal disease is a highly prevalent disease in the adult population. It is characterized by inflammation and progressive destruction of the periodontal tissues in response to specific microorganisms present in oral biofilm [7–10]. The pathogens associated with periodontal disease are frequently present in the human subgingival microbiota and are represented mainly by anaerobic gram-negative bacteria [11]. *A. actinomycetemcomitans*, Pasteurellaceae family, is a coccobacillus, fermentative, gram-negative, capnophilic, non-motile, and non-sporulating microorganism. This bacterium is considered the main etiological agent of localized aggressive periodontitis lesions, but is also associated with chronic periodontitis [12–18]. The progression of periodontal disease is associated with the virulence of the microorganism, together with the susceptibility of the host [19]. There are several virulence factors of *A. actinomycetemcomitans* that collaborate for its pathogenicity in periodontitis [20]. Leukotoxin, cytolethal distending toxins, bacteriocins, adhesins and lipopolysaccharide correspond to the variety of the microorganism virulence factors that may be associated with the pathogenesis of localized aggressive periodontitis [21]. These virulence factors attributed to *A. actinomycetemcomitans* are responsible for interacting with the host cells triggering an inflammatory response in the tissues supporting the teeth [22].

Fibroblasts and epithelial cells are the first cells to be activated in the oral cavity in response to exotoxic and endotoxic virulence factors of *A. actinomycetemcomitans*, performing an essential role in the production of cytokines involved in the inflammatory process. After this first local colonization, leukocytes (mainly monocytes and neutrophils) and dendritic cells are recruited to the site of infection giving sequence on inflammatory response [22, 23].

Recently, in vivo studies have demonstrated that β-glucans from *S. cerevisiae* present regulatory activity toward metabolism [24] and also modulate the expression of cycloxygenase-2 (COX-2), receptor activator of nuclear factor kappa-B ligand (RANK-L), and osteoprotegerin (OPG), decreasing alveolar bone loss caused by induced periodontal disease (ligature) in normal and diabetic animals [25]. However, knowledge of the molecular and biochemical mechanisms involved in β-glucan activity in periodontal disease is still not understood, demanding further research with advanced tissue culture techniques, examining the microbiota-host interaction. In that sense, the dual chamber model is an interesting in vitro model that mimics the human periodontum. It is constructed using a monolayer of epithelial keratinocytes and a subepithelial layer of fibroblasts on which the invasive periodontopathogen can be applied [26].

Thus, this study aims to evaluate the effects of β-glucan on the expression of inflammatory mediators and the metabolomic profile of oral cells using a dual-chamber model of epithelial and subepithelial cells infected by *A. actinomycetemcomitans.*

## Methods

### Bacterial strain and cells

*A. actinomycetemcomitans* strain (D7S-1) [27], human gingival epithelial cells (keratinocyte OBA-9) [28, 29] and human gingival fibroblast - HGF-1(ATCC® CRL-2014) were used in the present study.

### β-Glucan

The β-glucan utilized was the glucan from baker’s yeast *S. cerevisiae* (Sigma-Aldrich; St. Louis, MO), with a purity of 98%. Sterilized deionized water was used as the vehicle for β-glucan dilution.

### Antimicrobial activity

As a preliminary step, the antimicrobial activity and cytotoxicity of β-glucan were tested in order to determine the subsequent doses in the dual-chamber model. Antimicrobial activity was evaluated in *A. actinomicetemcomitans* after 24 h of treatment. Microorganisms were inoculated (1 × 10^6^ cfu/mL – colony-forming units per milliliter) in a 96-well microtiter plate with Trypticase Soy Broth (TSB; Becton Dickinson, Franklin Lakes, NJ) and β-glucan was immediately added in various concentrations (0 as control, and then subsequently from 1 μg/mL to 100 μg/mL) to determine the minimum inhibitory concentration (MIC) [30]. Microplates were maintained in a humidified incubator at 37 °C and 5% CO_2_. Microplates were maintained in a humidified incubator at 37 °C and 5% CO_2_. After 24 h, the contents of the wells were inoculated in Petri dishes with Trypticase Soy Agar (TSA; Becton Dickinson, Franklin Lakes, NJ) and incubated for 3 days. After this period, the cfu/mL was determined.

### Cytotoxicity assay

The in vitro cytotoxic effect was measured by the fluorometric resazurin method [31]. OBA-9 or HGF-1cells, cultured in DMEN medium (Lonza,Walkersville, MD) with10% of Fetal Bovine Serum - FBS (Lonza, Walkersville, MD), were seeded (1 × 10^5^ cells/mL) in a 96-well microtiter plate and maintained in a humidified incubator at 37 °C and 5% CO_2_. After 24 h, cell morphology was observed under an inverted microscope (EVOS FL; Life Technologies, Carlbad, CA) to confirm their adherence to the wells and to note their morphological changes. β-glucan (1–100 μg/mL) was added to the cell culture and incubated at 37 °C and 5% CO_2_. After 24 h, the medium was discarded, cells were washed with warm PBS (Lonza, Walkersville, MD), and replenished with fresh medium containing resazurin (Cell Titer Blue Viability Assay; Promega Corp, Madison, WI) [32]. Subsequently the plate was incubated at 37 °C and 5% CO_2_. After 4 h, the contents of the wells were transferred to a new microplate and the fluorescence was read in a microplate reader (SpectraMaxM5; Molecular Devices Sunnyvale, CA) with excitation at 550 nm, emission at 585 nm, and a cut off of 570 nm.

### Dual-chamber assay

**Fig. 1:**
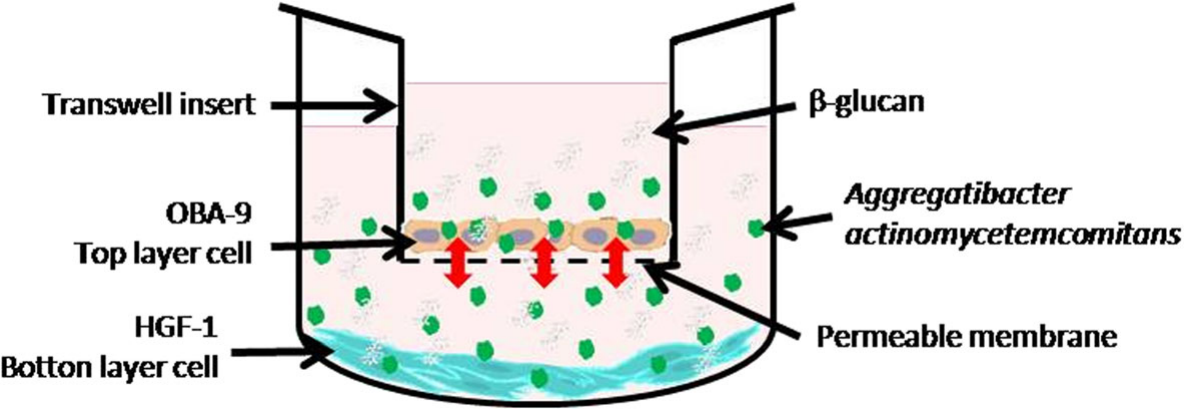
Dual-chamber model containing OBA-9 cell (keratinocytes) in the top layer (transwell insert) and HGF-1 cell (fibroblasts) in the bottom layer inoculated with *A. actinomicetemcomitans* and treated with different doses of β-glucan

The immunological effects of β-glucan were investigated using a dual-chamber model to mimic the periodontum (Fig. 1). Transwell inserts (8 μm pore × 0.3 cm^2^ of culture surface; Greiner Bio-One, Monroe, NC) were situated in a 24-well plate and OBA-9 cells (1 × 10^5^) were seeded intranswell inserts. HGF-1cells (1 × 10^5^) were seeded in the basal chamber. The plates were incubated at 37 °C in humid air containing 5% CO_2_ for 24 h. The trans-epithelial electric resistance (TEER) of each cell layer was measured with a Millicell-ERS volt-ohm meter (Millipore, Bedford, MA). Cell layer confluence in the Transwell insert was measured daily until optimal TEER was reached (>150 Ohm/cm^2^) which was found on the second day, when the medium in the basal chamber and insert were replaced with new medium (DMEN) containing *A. actinomicetemcomitans* (1 × 10^6^ cfu/mL). Medium containing the microorganism was added to the insert, passing through the upper layer of cells (OBA-9) and reaching the bottom cell layer (HGF-1) [26]. Immediately after inoculation of the dual-chamber with *A. actinomicetemcomitans* the β-glucan treatments (10 μg/mL or 20 μg/mL) were added and the plate was incubated at 37 °C in humid air containing 5% CO_2_. The time of exposure of the microorganism to β-glucan was 24 h. Each experiment was repeated three times with two replicates per group (*n* = 6) and the experimental groups were divided as described in Table 1. The two doses used were determined from the results found in the antimicrobial activity and cytotoxicity assay.

**Table 1:** Treatment groups - experimental design

Groups	Treatment
Control	Model with ^a^Aa inoculated and no treatment
BG 10	Model with Aa inoculated and treated with 10 μg/mL of β-glucan
BG 20	Model with Aa inoculated and treated with 20 μg/mL of β-glucan

^a^ *Aa Aggregatibacter actinomicetemcomitans*

### Sample collection for analysis

After the treatment period, the liquid contents of the wells were collected and centrifuged at 1200 rpm for 10 min. Following centrifugation, the supernatant was stored at −80 °C for subsequent metabolomic analysis. The remaining cell layer on the surface of the inserts and of the plate wells were used for RNA isolation (OBA-9 and HGF-1 separately) for gene analysis in quantitative real-timePCR.

### Gene expression –quantitative real-time PCR

Total RNA was isolated according to the Qiagen RNeasyMini Kit Protocol (Qiagen; Valencia CA). Purity and quantity of RNA were measured in a NanoPhotometer P360 (Implen; Westlake Village). Total RNA was converted into single-stranded cDNA using a high-capacity reverse transcription kit (QuantiTect Reverse Transcription Kit; Qiagen; Valencia, CA). From the cDNA obtained, an array for evaluation of gene expression of inflammatory response by quantitative real-time PCR (Prime PCR Pathway Plate/Acute Inflammation Response; Bio-Rad, Hercules, CA), was performed. Based on the results of the array, five genes/primers were selected for detailed study: IL-1-α, IL-18, B-cell lymphoma-2 (BCL-2), E1A Binding Protein (EP300) and prostaglandin-endoperoxidesynthase-2 (PTGS-2) (QuantiTect Primer Assay - Qiagen; Valencia, CA). For the selected primers, QuantiTect SYBR Green PCR Kits (Qiagen;Valencia, CA) were used. The reaction product was quantified by relative quantification using GAPDH as a reference gene. Data from standard threshold cycle (TC) of the equipment in real time (CFX Connect-Bio-Rad; Hercules, CA) were calculated and interpreted using the scan tool data qPCR array. Analysis of the relative quantitation was done using the ^ΔΔ^Ct comparative method [33].

### Metabolome analysis

The cell culture supernatant contents of the wells were collected and centrifuged at 1200 rpm for 10 min, at room temperature. Then, the supernatant was properly stored and sent for analysis at West Coast Metabolomics Center (UC Davis Genome Center; Davis, CA) for subsequent metabolomic analysis.

The metabolites were separated by gas chromatography/mass spectrometry (Agilent 6890, Santa Clara, CA/Leco Pegasus IV, St. Joseph, MI) according to standard methodology. The metabolites found were submitted to a comparison software and compared with a standard library of metabolites. Subsequently the data were submitted to statistical analysis (West Coast Metabolomics Centre (UC Davis Genome Center; Davis, CA) [34].

### Statistical analysis

Statistical analyses were done using analysis of variance (ANOVA). When F values indicated significant interactions, these were unfolded between factors. The analyses were performed in the statistical program SISVAR [35] at a significance level of α = 0.05.

## Results

**Fig. 2:**
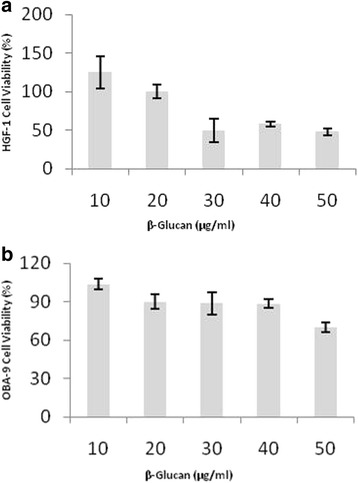
Cytotoxicity assay of cells treated with different doses of β-glucan: (**a**) OBA-9 cell (keratinocytes); (**b**) HGF-1 cell (fibroblasts). Cell viability was presented in percentage (%). *n* = 6

The antibacterial activity of β-glucan started at 10 μg/mL. Cytotoxicity assays were conducted in HGF-1 and OBA-9 cells and the results are shown in Fig. 2. The first concentration used was 10 μg/mL of β-glucan and resulted in 125% viability for HGF-1 and 104% for OBA-9 cells (Fig. 2a). The second concentration used was 20 μg/mL of β-glucan and resulted in 100% viability for HGF-1 and 90% for OBA-9 cells (Fig. 2b).

**Fig. 3:**
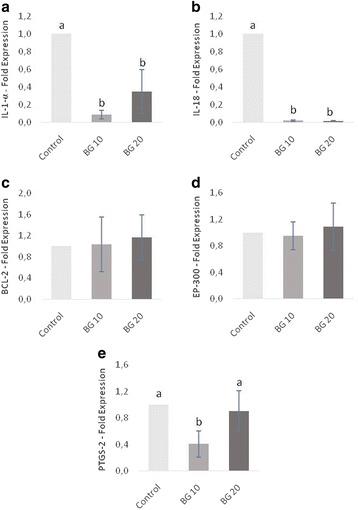
Relative expression of genes on HGF-1 cells (fibroblasts) by quantitative PCR: (**a**) interleukin 1 alpha (IL-1-α); (**b**) interleukin 18 (IL-18); (**c**) B-cell lymphoma 2 (BCL-2); (**d**) adenovirus E1A-associated 300 kDa protein (EP 300); (**e**) prostaglandin-endoperoxide synthase 2 (PTGS-2). Dual-chamber model inoculated with *A. actinomicetemcomitans* and treated with different doses of β-glucan. The control group has their mean expressed equal to 1 and treated groups have their mean relative to the control group. The results were expressed by mean followed standard deviation; *n* = 6 and *P* < 0.05

**Fig. 4:**
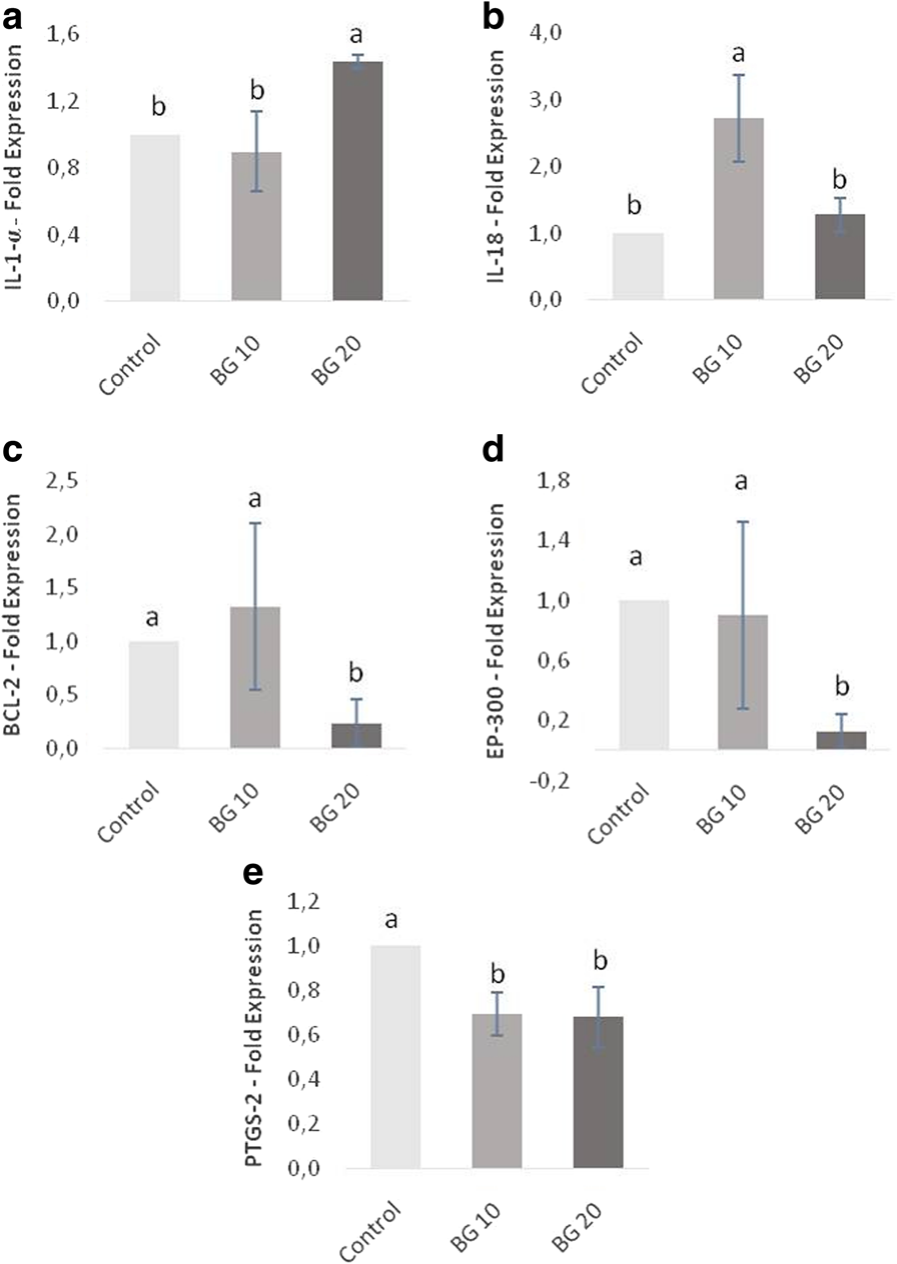
Relative expression of genes on OBA-9 cells (keratinocytes) by quantitative PCR: (**a**) interleukin 1 alpha (IL-1-α); (**b**) interleukin 18 (IL-18); (**c**) B-cell lymphoma 2 (BCL-2); (**d**) adenovirus E1A-associated 300 kDa protein (EP 300); (**e**) prostaglandin-endoperoxide synthase 2 (PTGS-2). Dual-chamber model inoculated with *A. actinomicetemcomitans* and treated with different doses of β-glucan. The control group has their mean expressed equal to 1 and treated groups have their mean relative to the control group. The results were expressed by mean followed standard deviation; *n* = 6 and *P* < 0.05

Quantitative real-time PCR results are presented in Figs. 3 and 4. Based on gene expression results of the inflammatory profile (acute inflammation response), five genes that showed greater variation in their expression (up or down regulation) were selected for detailed analysis: IL-1-α, IL-18, BCL-2, EP-300, and PTGS-2. The relative expression of IL-1-α (Fig. 3a) and IL-18 (Fig. 3b) genes in HGF-1 decreased with 10 μg/mL or 20 μg/mL of β-glucan in comparison with the control group (*p* < 0.05). In the same way, the expression of PTGS-2 (Fig. 3e) decreased with 10 μg/mL treatment; however, at a dose of 20 μg/mL it remained equal to that of the control group (*p* < 0.05). The expression of the BCL-2 (Fig. 3c) and EP-300 (Fig. 3d) genes were similar among groups.

For OBA-9, the expression of the IL-1-α (Fig. 4a) gene increased with 20 μg/mL and IL-18 (Fig. 4b) expression increased with 10 μg/mL (*p* < 0.05). The expression of BCL-2 (Fig. 4c), EP 300 (Fig. 4d), and PTGS-2 (Fig. 4e) decreased with the higher dose of β-glucan (*p* < 0.05).

**Fig. 5:**
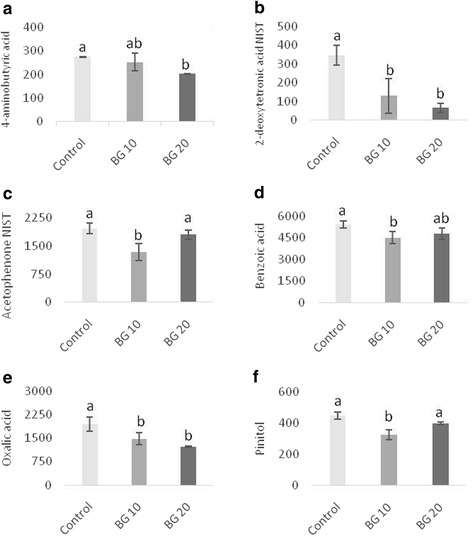
Metabolites obtained of cell culture supernatant (HGF-1 and OBA-9 co-culture cells). (**a**) 4-aminobutyric acid; (**b**) 2-deoxytetronic acid NIST; (**c**) acetophenone NIST; (**d**) benzoic acid; (**e**) oxalic acid; (**f**) pinitol. Dual-chamber model inoculated with *A. actinomicetemcomitans* and treated with different doses of β-glucan. The data is expressed in relative peak heights (mAU) from HPLC-MS analysis, which are unit-less (mean followed standard deviation); *n* = 4 and *P* < 0.05

The metabolomic study yielded a total of 283 metabolites, of which 120 were identified. Some metabolites presented significantly altered concentrations (Fig. 5). The 20 μg/mL β-glucan treatment presented lower (*p* < 0.05) concentrations of 4-aminobutyric acid (Fig. 5a). It was also observed that the β-glucan treatments used decreased (*p* < 0.05) the concentrations of 2-deoxytetronic acid NIST (Fig. 5b) and oxalic acid (Fig. 5e) at both concentration used (10 μg/mL and 20 μg/mL). Acetophenone NIST (Fig. 5c), benzoic acid (Fig. 5d) and pinitol (Fig. 5f) presented reduced (*p* < 0.05) concentrations when treated with only 10 μg/mL of β-glucan. All treatments were compared with the control group.

## Discussion

Human gingival fibroblasts represent the main cell type that form the soft connective tissues of the periodontium. These cells have a direct interaction with bacteria and their products [36], and perform an essential role in the production of cytokines involved during the inflammatory process [23]. The β-glucans present a capacity to stimulate the production of proinflammatory cytokines, thus modulating immune responses both specific and non-specific. Here, the authors extend further on their previous in vivo discovery [25] by showing the effects of β-glucans on gene expression of inflammatory cytokines and the metabolomic profile of mammalian cells.

For this study, the toxicity, anti-inflammatory activity, and effects on the transcriptome/metabolome of β-glucans on human cells were evaluated. The gene expression of IL-1-α and IL-18 in fibroblasts was reduced in the models treated with β-glucans. IL-1 is considered as a marker of periodontitis due to their involvement in the inflammation process (as inflammatory mediator) and its participation in the extracellular matrix and bone metabolism [37, 38]. In a study of experimental gingivitis, an increased concentration of IL-1 in gingival crevicular fluid was demonstrated [39]. The expression of IL-1-α and IL-1-β was induced in vitro from cultured gingival epithelial cells that were challenged with *A. actinomicetemcomitans* extracts [40]. These results indicate that gingival epithelial cells are the main source of these interleukins of the periodontium, which induce the production of additional inflammatory mediators [40]. IL-18 has pleiotropic action and participates in the innate and acquired immune responses [41], indicating a positive effect of β-glucan in reducing the expression of both IL-1-α and IL-18 in human fibroblasts. The decrease in these parameters may suggest an improvement in the inflammatory response associated with the immunomodulatory effects of β-glucans associated with their antimicrobial activity [3, 42–44]. Antagonistically, the expression of these same cytokines (IL-1-α and IL-18) observed in keratinocytes (OBA-9), indicated a result contrary to that seen in fibroblasts (HGF-1). Treatment with β-glucan increased the expression of IL-1-α and IL-18. This response may be due to a compensatory interaction between these different cell types. According to Di et al. [45], the expression of KGF (keratinocyte growth factor) and KGFR (keratinocyte growth factor receptor) observed in cocultures of keratinocytes and fibroblasts was influenced by the interaction of these different gingival cells. According to these authors, keratinocytes and fibroblasts can interact to dynamically regulate gene expression, what could have had such an effect on gingival cells conditions after treatment. In addition, the use of β-glucan decreased BCL-2 expression in keratinocytes. This protein exerts an antiapoptotic function, performing an essential role in the development of the immune response and tissue homeostasis [46].

β-glucan therapy regulated the expression of other immunomodulatory genes (EP300 and PTGS2), which shows an effect on more than one signaling pathway and can result in an important therapeutic effect. EP300, also known as p300, is involved in cell growth, proliferation, apoptosis, and embryogenesis [47, 48]. Some changes in its structure (derived from mutations) and the altered activity of this protein are linked with inflammation, malignant tumors, and developmental abnormalities [48]. Deng et al. [49] observed that p300 is involved in the stimulation of COX-2 expression induced by proinflammatory mediators. In the current study, treatment with β-glucan reduced EP300 expression in keratinocytes.

PTGS2, also known as COX-2, is an enzyme that is involved in the conversion of arachidonic acid to prostaglandins, performing an important role in the inflammatory response of periodontal tissues [50]. This enzyme has a preferentially inducible profile and is expressed by cells related to inflammatory processes [51] such as the response to inoculation by pathogenic micro-organisms. A recent study performed by our research group demonstrated lower COX-2 expression in diabetic rats with induced periodontal disease that were treated with β-glucan from *S. cerevisiae* [25]. Similarly, the present study showed a reduction in PTGS-2 expression, suggesting an improvement in the inflammatory profile as a function of treatment with β-glucan.

The metabolomic study in the present work explored the influence of β-glucan treatment on cell metabolic profile and found significant changes in 4-aminobutyric acid, 2-deoxytetronic acid NIST, oxalic acid, acetophenone NIST, benzoic acid, and pinitol. 4-aminobutyric acid, more commonly known as gamma-aminobutyric acid (GABA), is a non-protein amino acid that acts as the main inhibitory neurotransmitter of the central nervous system in animals and humans [52]. Some studies have linked increased intake of GABA or its analogs with multiple health benefits, for example, lowering blood pressure in hypertensive animals and humans [53–56]. In addition, studies indicate that GABA ingestion from enriched natural sources, has an inhibitory effect on the proliferation of cancer cells and has a enhancer action on cancer cell apoptosis [57]. Other compounds, such as benzoic acid and pinitol, are derived from plants and have multifunctional properties. Benzoic acid is an aromatic carboxylic acid present in the tissues of plants and animals and can also be produced by microorganisms [58]. Pinitol, also called D-pinitol, is a compound with multifunctional properties, among them, anti-inflammatory, cardioprotective, and anti-hyperlipidemicactions. Furthermore, pinitol is known to have properties similar to those of insulin [59–61].

A study compared the metabolomic profile of patients with different levels of gingival bleeding. Metabolomic analysis of this study indicated significant changes in the composition of metabolites, especially the short chain carboxylic acids propionate and n-butyrate, which tracked clinical changes in gingivitis severity [62]. Another study analyzed the metabolomic profile in saliva and plasma samples of diabetic patients with healthy periodontium, gingivitis and periodontitis. They observed increased levels of markers of cellular energetic stress, increased purine degradation and glutathione metabolism through increased levels of oxidized glutathione and cysteine-glutathione disulfide, markers of oxidative stress (guanosine and inosine), increased amino acid levels suggesting protein degradation, and increased ω-3 (docosapentaenoate) and ω-6 fatty acid (linoleate and arachidonate). According to the authors, these metabolites associated with the periodontal condition may be useful for developing diagnoses and therapeutics adapted to the diabetic population [63]. Thus, we believe that metabolomic profile analysis may be a useful tool in investigating of the β-glucans action on periodontal disease and the changes in metabolites can be used as markers of the disease.

The results observed in the present study demonstrated that the β-glucan was able to modulate gene expression and alter the concentrations of different metabolites by modifying the immune cell response to a challenge with *A. actinomicetemcomitans*. β-glucan treatment (10 μg/mL or 20 μg/mL) reduced the concentrations of 4-aminobutyric acid, 2-deoxytetronic acid NIST, oxalic acid, acetophenone NIST, benzoic acid, and pinitol. In fibroblasts (HGF-1), the relative expression of IL-1-α, IL-18, and PTGS-2 genes decreased with 10 μg/mL or 20 μg/mL of β-glucan. In keratinocytes (OBA-9), the expression of BCL-2, EP-300, and PTGS-2 decreased with the higher dose of β-glucan. Such genes are considered a marker for many dysfunctions, such as periodontal disease, due to their functions as inflammatory mediators. The modulation of gene expression these markers may indicate an improvement in inflammatory profile and a possible reduction in microbial activity.

## Conclusions

Treatment with β-glucans from *Saccharomyces cerevisiae* administered for 24 h in a dual-chamber model positively modulated the immune response and metabolites production.
